# High polygenic predisposition for ADHD and a greater risk of all-cause mortality: a large population-based longitudinal study

**DOI:** 10.1186/s12916-022-02279-3

**Published:** 2022-02-23

**Authors:** Olesya Ajnakina, Diana Shamsutdinova, Theresa Wimberley, Søren Dalsgaard, Andrew Steptoe

**Affiliations:** 1 Department of Biostatistics & Health Informatics, Institute of Psychiatry, Psychology and Neuroscience, King’s College London, 16 De Crespigny Park, London, SE5 8AF UK; 2 Department of Behavioural Science and Health, Institute of Epidemiology and Health Care, University College London, 1-19 Torrington Place, London, WC1E 7HB UK; 3 National Centre for Register-based Research, Department of Economics and Business, School of Business and Social Sciences, Aarhus University, Fuglesangs Allé 26, Building R, 8210 Aarhus V, Denmark; 4 The Lundbeck Foundation Initiative for Integrative Psychiatric Research, iPSYCH, Fuglesangs Allé 26, 8210 Aarhus V, Denmark; 5 Centre for Integrated Register-based Research (CIRRAU), Aarhus University, Nordre Ringgade 1, 8000 Aarhus C, Denmark; 6 Mental Health Services of the Capital Region, Department of Child and Adolescent Psychiatry, Glostrup, Kildegaardsvej 28, Opgang 3A, 1 sal, 2900 Hellerup, Denmark; 7 Institute of Clinical Medicine, University of Copenhagen, Nørregade 10, 1165 København, Denmark

**Keywords:** Attention deficit hyperactivity disorder, Mortality, Genome-wide association studies, Polygenic predisposition, Healthy ageing

## Abstract

**Background:**

Attention deficit hyperactivity disorder (ADHD) is a highly heritable, neurodevelopmental disorder known to associate with more than double the risk of death compared with people without ADHD. Because most research on ADHD has focused on children and adolescents, among whom death rates are relatively low, the impact of a high polygenic predisposition to ADHD on accelerating mortality risk in older adults is unknown. Thus, the aim of the study was to investigate if a high polygenetic predisposition to ADHD exacerbates the risk of all-cause mortality in older adults from the general population in the UK.

**Methods:**

Utilising data from the English Longitudinal Study of Ageing, which is an ongoing multidisciplinary study of the English population aged ≥ 50 years, polygenetic scores for ADHD were calculated using summary statistics for (1) ADHD (PGS-ADHD_single_) and (2) chronic obstructive pulmonary disease and younger age of giving first birth, which were shown to have a strong genetic correlation with ADHD using the multi-trait analysis of genome-wide association summary statistics; this polygenic score was referred to as PGS-ADHD_multi-trait_. All-cause mortality was ascertained from the National Health Service central register that captures all deaths occurring in the UK.

**Results:**

The sample comprised 7133 participants with a mean age of 64.7 years (SD = 9.5, range = 50–101); of these, 1778 (24.9%) died during a period of 11.2 years. PGS-ADHD_single_ was associated with a greater risk of all-cause mortality (hazard ratio [HR] = 1.06, 95% CI = 1.02–1.12, *p* = 0.010); further analyses showed this relationship was significant in men (HR = 1.07, 95% CI = 1.00–1.14, *p* = 0.043). Risk of all-cause mortality increased by an approximate 11% for one standard deviation increase in PGS-ADHD_multi-trait_ (HR = 1.11, 95% CI = 1.06–1.16, *p* < 0.001). When the model was run separately for men and women, the association between PGS-ADHD_multi-trait_ and an increased risk of all-cause mortality was significant in men (HR = 1.10, 95% CI = 1.03–1.18, *p* = 0.003) and women (HR = 1.11, 95% CI = 1.04–1.19, *p* = 0.003).

**Conclusions:**

A high polygenetic predisposition to ADHD is a risk factor for all-cause mortality in older adults. This risk is better captured when incorporating genetic information from correlated traits.

**Supplementary Information:**

The online version contains supplementary material available at 10.1186/s12916-022-02279-3.

## Background

Attention deficit hyperactivity disorder (ADHD) is a neurodevelopmental disorder, characterized by age-inappropriate inattention, overactivity or impulsiveness [1]. One of the important outcomes related to ADHD diagnosis is a significantly higher risk of dying compared to adults who never received a diagnosis of this disorder [2, 3]. Although evidence suggests that accidents, substance use disorders, criminality and extensive tobacco use may contribute to the excess of deaths in people with a diagnosis of ADHD [2–4], accounting for these factors does not fully reduce the risk of early death [2]. This indicates that there are other underlying factors driving the nexus of ADHD and mortality.

ADHD is a highly heritable disorder [5], with a pooled analysis of 20 twin studies reporting a mean heritability estimate of 76% [6]. More recent genome-wide association studies of ADHD revealed that the genetic architecture of this disorder is characterised by multiple common genetic markers spread across the entire genome [7]. Building on the results from genome-wide association studies, polygenic score (PGS), which measures an individual genetic predisposition to a trait by combining the effects of many common genetic variants associated with it [8], confirmed that ADHD is highly polygenic in nature [9].

However, the impact of having a higher polygenic predisposition to ADHD extends beyond ADHD diagnosis. Indeed, a high polygenic predisposition to ADHD was shown to associate with higher levels of symptoms related to ADHD diagnosis, such as inattention, hyperactivity and impulsivity, in children and adolescence [10]. Similarly, it was shown to influence ADHD traits in the general population [11] including injuries and emergency ward visits in early childhood [12] and conduct problems in childhood in people without ADHD diagnosis [11]. Based on this accumulating evidence, it may be argued that a higher polygenetic predisposition to ADHD may also exacerbate the risk of mortality in the adults without ADHD diagnosis. However, because most research in ADHD has focused on children and adolescents, among whom death rates are relatively low [3], the magnitude of impact of having a high polygenic predisposition to ADHD on accelerating mortality risk in older adults is unknown.

PGSs can further be used to assess predisposition to a condition that may never be expressed phenotypically, highlighting a shared genetic risk between traits and health conditions [13, 14]. For instance, PGS analyses have revealed shared polygenic contributions between ADHD diagnosis and conduct problems [15], schizophrenia [16], major depressive disorders [17] and other neurodevelopmental traits [11] in the general population. Genetic correlations were also shown between ADHD diagnosis and several other traits, such as educational attainment, age at first birth, neuroticism, and physical health conditions, including body mass index and Alzheimer disease [18]. It, therefore, may be argued that to fully capture the impact of polygenetic influences on mortality risk, the genetic information from the traits that correlate with ADHD diagnosis may need to be incorporated into the PGS [19]. This in turn may provide more detailed insights into the genetic make-up of mortality informing the search for its biological mechanisms.

Therefore, drawing on a large, phenotypically well-defined sample of population-representative older adults without a known diagnosis of ADHD, the aim of the present study was to investigate the extent to which the polygenic predisposition to ADHD is associated with all-cause mortality in general population in the UK. We further investigated if utilising a measure of polygenic predisposition to ADHD based on multiple traits that have an overlapping genetic make-up with ADHD provides a stronger predictor of the nexus of ADHD and mortality. We hypothesised that there would be a significant association between polygenic score for ADHD and all-cause mortality during the 11-year follow-up period in general population in the UK. We further hypothesised that a polygenic predisposition to ADHD based on multiple overlapping traits will provide a significantly stronger predictor of the nexus of ADHD and mortality compared to polygenic score capturing polygenic predisposition to ADHD diagnosis only.

## Methods

### Study design and participants

We utilised data from the English Longitudinal Study of Ageing (ELSA), which is an ongoing large, multidisciplinary study of a nationally representative sample of the English population aged ≥ 50 years [20]. The ELSA study started in 2002–2003 (wave 1) with participants recruited from the Health Survey for England, which was designed to monitor the health of the general population, who were then followed up every 2 years. The ELSA sample is periodically refreshed with younger participants to ensure that the full age spectrum is maintained [20]. Compared with the national census, the ELSA sample has been shown to be representative of the non-institutionalised general population aged ≥ 50 residing in the UK [20]. Because the bloods (for genetic data) were collected by nurses during home visit at wave 2 (2004–2005) for the core members who started at wave 1 and wave 4 (2008–2009) for the participants joining the study at wave 4 through the refreshment sample, the data from these waves formed our baseline. None of the participants included in the analyses had a self-report participant’s physician diagnosis of ADHD. Because the construction of PGSs is dependent on availability of summary statistics from genome-wide association studies (GWAS), which are largely based on population of European descent [21], we removed participants with ancestral admixture (Additional file 1: Figs S1-2). Compared to respondents who were excluded from analyses, our final analytical sample encompassed a higher proportion of responders with a higher educational attainment, higher accumulated wealth, lower proportion of people with depressive symptoms and a lower proportion of smokers (Additional file 1: Table S1).

### Outcome

The outcome was all-cause mortality that occurred from baseline till the end of wave 8 (2016–2017) encompassing an average follow-up period of 11.2 years (SD = 3.1, median = 13.3, range = 1.0–13.7, 79829.8 person-years). The rate of mortality recorded at each year of follow-up is presented in Additional file 1: Table S2. All-cause mortality was ascertained from the National Health Service central register that captures all deaths occurring in the UK. All participants included in this study provided written consent for the linkage to their official records. Survival time was defined as the period from baseline when all ELSA participants were alive to the date when an ELSA participant was reported to have died during the follow-up period. For those who did not die during follow-up, the survival time was calculated using the period spanning from baseline until the end of wave 8 (2016–2017).

### Genetic data

The genetic data were extracted from the blood draws taken during home visits. The genome-wide genotyping was performed at University College London Genomics in 2013–2014 using the Illumina HumanOmni2.5 BeadChips (HumanOmni2.5-4v1, HumanOmni2.5-8v1.3), which measures ~ 2.5 million markers that capture the genomic variation down to 2.5% minor allele frequency (MAF).

### Quality control

Single-nucleotide polymorphism (SNPs) were excluded if they were non-autosomal, MAF was < 1%, if more than 2% of genotype data were missing and if the Hardy-Weinberg Equilibrium *p* < 10^−4^. Samples were removed based on call rate (< 0.99), suspected non-European ancestry, heterozygosity and relatedness and if the recorded sex phenotype was inconsistent with genetic sex (Additional file 1: Table S3). To investigate population structure, principal components analysis was conducted [22, 23] in PLINK 1.9 [24]. We retained 10 principal components to account for any ancestry differences in genetic structures that could bias results [22, 23].

### Polygenic score (PGS)

To calculate PGS for ADHD (PGS-ADHD), summary statistics from several large genome-wide association studies (GWAS) of ADHD including the one conducted in the 23andMe with a combined sample of 117754 participants [9, 25] were used. PGS-ADHD was calculated employing LDpred [26], which was applied to HapMap3 SNPs as the external linkage disequilibrium (LD) reference sample. This method assumes that SNP effects are drawn from mixtures of distributions with the key parameters defining these architectures estimated through Bayesian framework [26]. LDpred is one of the robust approaches that maximises the power of PGSs [27]; we refer to this PGS as PGS-ADHD_single_.

To calculate PGS for ADHD based on multiple traits that have an overlapping genetic make-up with ADHD, the Multi-Trait Analysis of GWAS (MTAG) approach was employed [19]. MTAG was chosen because it can be applied to GWAS summary statistics (without access to individual-level data) from an arbitrary number of traits. Because many GWAS summary statistics are likely to have overlapping samples, to account for (possibly unknown) sample overlap between the GWAS results for different traits MTAG uses bivariate LD score regression [19]. Here, genetic correlations between ADHD and 52 traits related to physical and mental health, behaviours, personality types and educational attainment were estimated using LD score regressions [25]. These analyses showed that there were two genetic correlations that were sufficiently strong to be included in the multi-trait PGS for ADHD; these correlations were between ADHD and with chronic obstructive pulmonary disease (*r*^2^ = 0.65) and age at birth of first child (*r*^2^ = − 0.62) [25]. MTAG then conducted meta-analysis of these GWAS summary statistics for these three traits generating multi-trait summary statistics for ADHD, which in turn was used to calculate PGS-ADHD_multi-trait_ using LDpred as described above.

Because calculating PGS based on clumping and thresholding method (PC+T, also known as P+T or C+T) as implemented in PRSice [28] is often used as the benchmark method [27], we carried out sensitivity analyses. Specifically, we calculated PGS-ADHD using PC+T in PRSice [28] and employing the publicly available summary statistics from a GWA meta-analysis, which encompassed 20183 individuals diagnosed with ADHD and 35191 controls [9]. Therefore, this set of analyses utilised a different computational approach for calculating PGS for ADHD and the commonly publicly available summary statistics of GWA meta-analysis of individuals diagnosed with ADHD and controls [9]. Here, PGSs are calculated as a weighted sum of the allele dosages, summing over the common markers abiding by the *p*-value thresholds (*p*_Ts_) (i.e. 0.001, 0.01, 0.05, 0.1, 0.3, and 1) weighted according to the strength of effect estimate. Because a PGS at *p*-value thresholds *p*_T_ = 1 was recommended as the optimal threshold for further analyses [29], we utilised PGS-ADHD that was based on *p*_T_ = 1 assuming all genetic markers contributed to ADHD diagnosis; we refer to this PGS as PGS-ADHD_*P*T = 1_. Nonetheless, the results related to the remaining *p*_Ts_ are provided in the Additional file 1: Table S4. To aid interpretability of the results, PGS-ADHD_single_, PGS-ADHD_multi-trait_, and PGS-ADHD_*P*T = 1_ was standardized to a mean of 0 (SD = 1).

### Statistical analyses

#### Association analyses

To investigate the relationship between PGS-ADHD and mortality during the 11-year follow-up period, Cox proportional hazards regressions were conducted. To test the proportional hazards assumptions of the Cox models, the scaled Schoenfeld residual test was computed, and all models met the proportional hazards assumptions (all *p* > 0.05) (Additional file 1: Table S5). We included sex, age and genetic ancestry as quantified by the top four principal components [22] as covariates. Given the wide range of our participants’ ages, the impact of additional years from the baseline age may not have a stable linear increase for the log-hazards. Therefore, we tested the inclusion of polynomial age terms to the model. We found that adding age^2^ and age^3^ terms significantly improved the global fit of the Cox models. Indeed, global test on proportionate hazard assumption with normalised age only had *p* = 0.104, with age and age^2^ had *p* = 0.158, with age, age^2^ and age^3^ had *p* = 0.352. Consequently, to capture the non-linear effects of ageing, we further included age^2^ and age^3^ as covariates. We have additionally tested for the interaction effect between polygenic scores for ADHD and sex, and interaction between age and sex. As the former interaction effect was not significant, we did not include it in the models. The interaction between age and sex was significant; thus, we included it in the models.

Considering ADHD affects life expectancy differently in men and women [2], we ran all models separately for men and women. Additionally, to limit the overriding influence of age in a “cohort of survivors”, we re-ran all models limiting the sample to those who were aged ≤ 75 years old. We measured the predictive power of each polygenic score by its incremental *R*^2^ value, defined as the increase in *R*^2^ as we moved from a cox regression including a set of covariates (age, age^2^ and age^3^, sex, interaction between age and sex, first 4 principal components of the genetic data) to a regression that additionally included PGS as an independent variable; this procedure was repeated for each PGS separately. All analyses were carried out in RStudio version 4.0.3 [30]; all tests for analyses were two-tailed; *p* ≤ 0.05 were considered statistically significant.

## Results

### Study participants

Baseline sample characteristics of ELSA participants are presented in Table 1. The sample comprised 7133 individuals who were representative of European-ancestry older adults with the baseline mean age for the entire sample of 64.7 years (standard deviation (SD) = 9.5, median = 63.0, IQR = 14, range = 50–101); 46.2% (*n* = 3294) were men and 84.9% (*n* = 6059) were ≤ 75 years old at baseline. Of the entire sample, 1778 (24.9%) died during the 11-year follow-up with an average length of survival of 134.3 months (SD = 37.5, median = 156, IQR = 46). Compared to those who remained alive at the end of follow-up, a higher proportion of responders who died during the follow-up period was smokers (15.0% vs. 19.8%, *x*^2^_1_ = 22.4, *p* < 0.001) and not married (27.0% vs. 44.5%, *x*^2^_1_ = 190.3, *p* < 0.001); the latter group further tended to report severe depressive symptoms (12.6% vs. 18.4%, *x*^2^_1_ = 37.1, *p* < 0.001), low accumulated wealth (30.7% vs. 45.7%, *x*^2^_2_ = 151.9, *p* < 0.001) and a lower educational attainment (mean = 14.4 vs. mean = 12.9 years, *t*_7131_ = 14.1, *p* < 0.001).

**Table 1:** Baseline sample characteristics of ELSA participants

Baseline characteristics			Mortality						
	Total sample		No		Yes		Test statistics		
	***n*** = 7133		***n*** = 5355 (75.1%)		***n*** = 1778 (24.9%)				
	Mean/*n*	SD/%	Mean/*n*	SD/%	Mean/*n*	SD/%	*t*/x^2^	df	*p*
Age (years)	64.7	9.5	61.8	7.6	73.4	9.4	-62.3	7131	< 0.001
Median (IQR)	63.0	14.0	60.0	11.0	74.0	13.0			
Sex									
Men	3294	46.2	2361	44.1	933	52.5	37.78	1	< 0.001
Women	3839	53.8	2994	55.9	845	47.5			
Currently a smoker	1152	16.2	801	15.0	351	19.8	22.4	1	< 0.001
Accumulated wealth									
High	2428	34.9	1987	38.1	441	25.1	151.9	2	< 0.001
Intermediate	2137	30.7	1624	31.2	513	29.2			
Low	2401	34.5	1598	30.7	803	45.7			
Relationship status									
Not married	2239	31.4	1447	27.0	792	44.5	190.3	1	< 0.001
Currently married	4894	68.6	3908	73.0	986	55.5			
Severe depressive symptoms	998	14.0	673	12.6	325	18.4	37.1	1	< 0.001
Frequent alcohol intake	4341	64.7	3426	66.9	915	57.7	45.2	1	< 0.001
Limiting longstanding illness	3917	54.9	2692	50.3	1225	68.9	187.1	1	< 0.001
Educational attainment years	14	3.8	14.4	3.8	12.9	3.7	14.1	7131	< 0.001
Survival time, month	134.3	37.5	148.9	19.7	90.4	43.7	77.0	7131	< 0.001
Median (IQR)	156.0	46.0	158.0	6.0	92.5	72.0			
Length of follow-up, years	11.2	3.1	12.4	1.6	7.5	3.6	77.0	7131	< 0.001
Median (IQR)	13.0	3.8	13.2	0.5	7.7	6.0			

*df* degrees of freedom, *SD* standard deviation, *IQR* interquartile range which shows a difference between 0.75 and 0.25 quantiles

### PGS-ADHD_single_ and risk for all-cause mortality

**Fig. 1:**
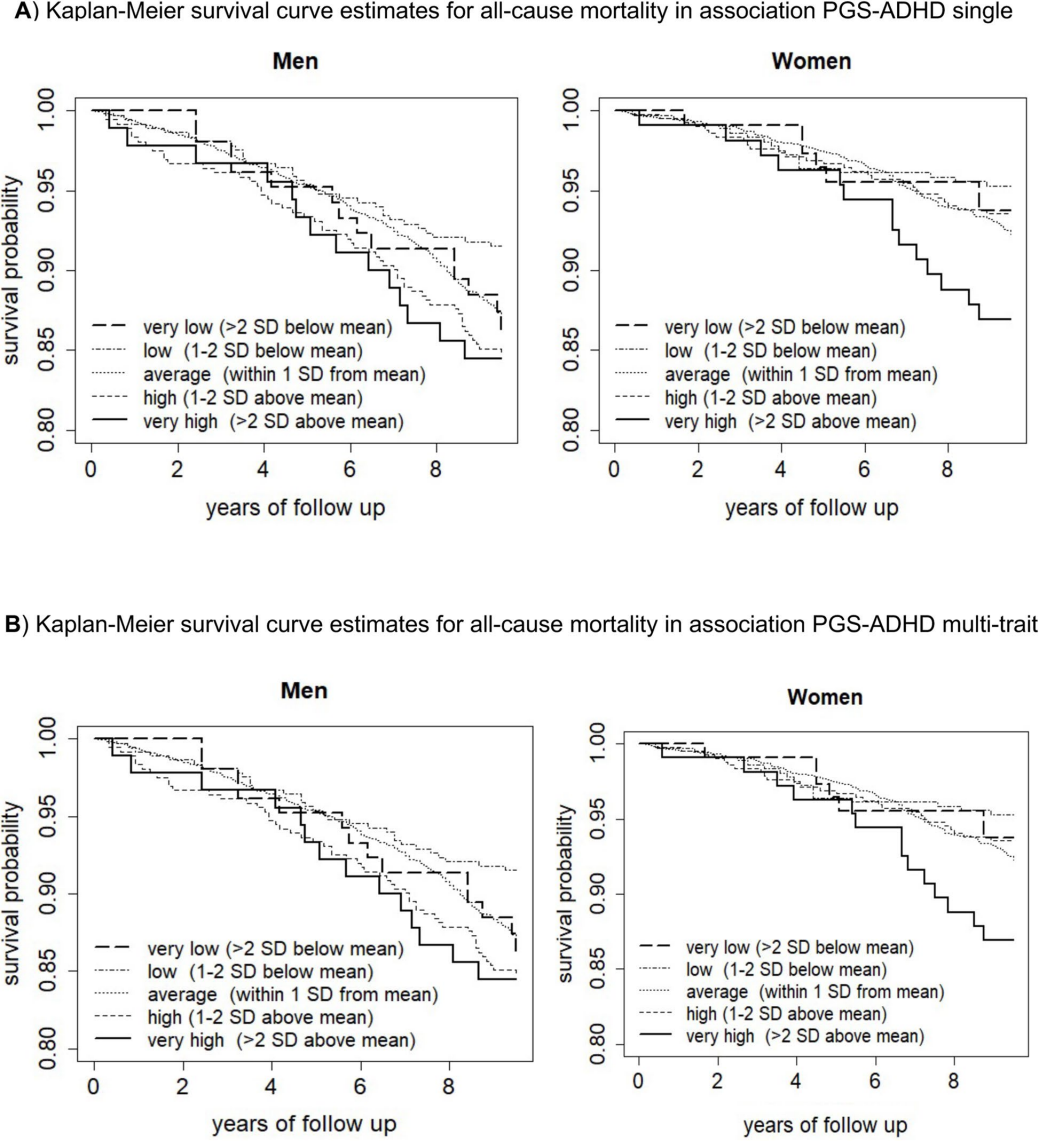
Kaplan-Meier survival curve estimates for all-cause mortality over a follow-up period in association with different polygenic scores for ADHD

PGS-ADHD_single_ was associated with a greater risk for all-cause mortality during follow-up in the entire sample (hazard ratio [HR] = 1.06, 95% CI = 1.02–1.12, *p* = 0.010, *R*^2^ = 0.07%) (Table 2). Further analyses showed that this association was significant in men (HR = 1.07, 95% CI = 1.00–1.14, *p* = 0.043, *R*^2^ = 0.09%) (Table 2, Fig. 1A). When analyses were limited to adults who were aged 50–75 years old, for every SD increase in PGS-ADHD_single_, the risk for all-cause mortality in men increased by 12% (HR = 1.12, 95% CI = 1.03–1.22, *p* = 0.006, *R*^2^ = 0.24%), whereas the relationship of PGS-ADHD_single_ and all-cause mortality remained insignificant in women.

**Table 2:** Cox regression analyses highlighting associations between PGD-ADHD_single_ and risk for all-cause mortality during a follow-up period

Polygenic scores for ADHD	The whole sample				Men				Women			
	HR	95% CI	***p***	***R***^**2**^	HR	95% CI	***p***	***R***^**2**^	HR	95% CI	***p***	***R***^**2**^
Participants all ages												
PGD-ADHD_single_	1.06	1.02–1.12	0.010	0.07%	1.07	1.00–1.14	0.043	0.09%	1.06	0.99–1.13	0.121	0.05%
Participants aged 50–75 years at start												
PGD-ADHD_single_	1.09	1.03–1.17	0.006	0.11%	1.12	1.03–1.22	0.006	0.24%	1.05	0.96–1.16	0.284	0.03%

Model was adjusted for baseline age, sex, interaction between age, sex and first 4 PCs to adjust for genetic ancestry; to capture non-linear effects of ageing, we included age^2^ and age^3^ as covariates

*ADHD* attention deficit hyperactivity disorder, *HR* hazard ratio, *CI* confidence intervals

### PGS-ADHD_multi-trait_ and risk for all-cause mortality

Risk for all-cause mortality increased by an approximate 11% for each increase in SD in PGS-ADHD_multi-trait_ (HR = 1.11, 95% CI = 1.06–1.16, *p* < 0.001, *R*^2^ = 0.07%) (Table 3). When the model was run separately for men and women, this association between PGS-ADHD_multi-trait_ and the increased risk for all-cause mortality was significant in men (HR = 1.10, 95% CI = 1.03–1.18, *p* = 0.003, *R*^2^ = 0.09%) and women (HR = 1.11, 95% CI = 1.04–1.19, *p* = 0.003, *R*^2^ = 0.05%) (Table 3, Fig. 1B). When analyses were limited to adults who were aged 50–75 years old, every SD increase in PGS-ADHD_multi-trait_ was associated with an increase in the risk for all-cause mortality by 14% in men (HR = 1.14, 95% CI = 1.05–1.25, *p* = 0.002, *R*^2^ = 0.24%) and 13% in women (HR = 1.13, 95% CI = 1.03–1.24, *p* = 0.014, *R*^2^ = 0.03%).

**Table 3:** Cox regression analyses highlighting associations between PGS-ADHD_multi-trait_ and risk for all-cause mortality during a follow-up period

Polygenic scores for ADHD	The whole sample				Men				Women			
	HR	95% CI	***p***	***R***^**2**^	HR	95% CI	***p***	***R***^**2**^	HR	95% CI	***p***	***R***^**2**^
Participants all ages												
PGS-ADHD_multi-trait_	1.11	1.06–1.16	< 0.001	0.07%	1.10	1.03–1.18	0.003	0.09%	1.11	1.04–1.19	0.003	0.05%
Participants aged 50–75 years at start												
PGS-ADHD_multi-trait_	1.14	1.07–1.21	< 0.001	0.11%	1.14	1.05–1.25	0.002	0.24%	1.13	1.03–1.24	0.014	0.03%

Model was adjusted for baseline age, sex, interaction between age, sex and first 4 PCs to adjust for genetic ancestry; to capture non-linear effects of ageing, we included age^2^ and age^3^ as covariates

*ADHD* attention deficit hyperactivity disorder, *HR* hazard ratio, *CI* confidence intervals

### Sensitivity analyses

When we repeated the cox regression analyses using PGS-ADHD_*P*T = 1_, the results were closely aligned with those observed for PGS-ADHD_single_ (Additional file 1: Table S4). Indeed, there was an association between PGS-ADHD_*P*T = 1_ and a greater risk for all-cause mortality in the entire sample (HR = 1.05, 95% CI = 1.00–1.10, *p* = 0.047, *R*^2^ = 0.001%), which was shown to be significant in men (HR = 1.07, 95% CI = 1.00–1.14, *p* = 0.047, *R*^2^ = 0.09%) and not women (HR = 1.03, 95% CI = 0.96–1.10, *p* = 0.416, *R*^2^ = 0.01%). When analyses were limited to adults who were aged 50–75 years old, for every SD increase in PGS-ADHD_*P*T = 1_, the risk for all-cause mortality in men increased by 11% (HR = 1.11, 95% CI = 1.02–1.21, *p* = 0.014, *R*^2^ = 0.19%), though the relationship remained insignificant in women.

## Discussion

To our knowledge, this is the first study to investigate the relationships of a polygenic predisposition to ADHD, as measured with a single trait and multi-trait approaches, with a risk of all-cause mortality among the nationally representative adults aged ≥ 50 years without known diagnosis of ADHD, who were followed over an average period of 11 years. Cumulatively, our results contribute to a better understanding of the role a higher polygenic predisposition to ADHD plays in accelerating risk of death in the general population in the UK, for which knowledge is currently lacking.

Assuming a variation in risk for mortality is a function of the degree of a polygenic predisposition to ADHD, our results showed that the risk of all-cause mortality is amplified by a higher loading of common genetic markers associated with ADHD diagnosis, with a greater genetic liability indicating a greater risk of all-cause mortality. This finding supports the results from epidemiological studies suggesting a link between ADHD and an increased mortality risk among adults [2, 3]. This results further highlights the existence of a specific subpopulation that may be at a higher risk of all-cause mortality based on their polygenetic loading who may benefit from the prioritisation for screening programmes, lifestyle modifications or preventive treatments. Nonetheless, because PGS-ADHD_single_ was built using results from large-scale biomedical databases, such as UK Biobank and 23andMe, the use of this PGS may be limited as these data sources do not tend to be publicly available. When we repeated the analyses using PGS-ADHD_*P*T = 1_ which was calculated using the commonly available summary statistics of individuals diagnosed with ADHD and controls [9], these results remained largely unchanged. This suggests that while summary statistics that combine genetic information from large-scale biomedical databases, such as UK Biobank and 23andMe, might have increased power to detect genetic variants associated with ADHD of large effects [8], these data sources may not be necessary to retain power of polygenic scores for this disorder.

Our further analyses revealed that this relationship of a polygenic predisposition to ADHD with a higher risk of all-cause mortality was specific to men, where one standard deviation increase in PGS-ADHD_single_ was associated with an increased risk of all-cause mortality by an average 6%; the observed risk doubled among men who were age ≤ 75 years old. The significant association between the PGS-ADHD and all-cause mortality risk observed in men, which did not reach the standard level of significance in women, may imply that men may particularly be vulnerable to the negative impact of having a higher aggregate of loci for ADHD in relation in the increased risk for all-cause mortality. In epidemiological research, the nexus of ADHD diagnosis and mortality has been linked to an increased risk of criminal and antisocial activities [2, 31], substance abuse disorders, violence and fatal traffic accidents [2, 3, 32, 33]. Our results may suggest that these risky and less prudent behaviours, are, at least in part, influenced by common genetic variants associated with ADHD diagnosis, which in turn is associated with a greater risk of all-cause mortality in men [3].

Nonetheless, mortality has been shown to have shared polygenic contributions with several other traits and conditions beyond ADHD, including educational attainment [34], coronary artery disease [35] and type 2 diabetes [36]. We extend these findings by showing that ADHD has a shared genetic component with chronic obstructive pulmonary disease and younger age of giving first birth [25, 37]. The link between ADHD and respiratory-related diseases, such as asthma and chronic obstructive pulmonary disease is quite robust. For example, a recent study encompassing 4,789,799 individuals residing in Sweden showed that genetic factors explained 60–69% of correlations between ADHD diagnosis and respiratory-related [37]. While advanced societies have experienced a rapid postponement of age of giving first birth [38], people with a diagnosis of ADHD are still more likely to have teenage pregnancy compared to individuals without this diagnosis [39], which is associated with a two-fold increased risk of ADHD in the offspring [40, 41]. Our study is the first to show that a shared genetic liability between ADHD, chronic obstructive pulmonary disease, and younger age of giving first birth is further linked to the all-cause mortality in older men and women without ADHD diagnosis.

Indeed, utilising the genetic data from these traits into a polygenic score (i.e. PGS-ADHD_multi-trait_) showed that the nexus of ADHD diagnosis and all-cause mortality was equally strong in men and women. Thus, it may be argued that by focusing on a single trait, the standard methods for calculating PGS do not capture the full impact of genetic predisposition to ADHD [18], which in turn masks potential relationships with all-cause mortality in the general population of older adults. Cumulatively, our results suggest that older men and women who are susceptible to either ADHD or correlated traits are more susceptible to all-cause mortality. Although the effect sizes for ADHD_multi-trait_ were higher compared to the ADHD_single_, the predictive power of each polygenic score, as was measured by an incremental *R*^2^ value, was the same for ADHD_multi-trait_ and ADHD_single_ highlighting that increase in predictive power in the ADHD_multi-trait_ was not significant. Nonetheless, this mapping of aetiological sources of cross-disorder overlap can guide future research aiming to identify specific mechanisms contributing to risk of ADHD.

### Strengths and limitations

We analysed a large, longitudinal and population-based cohort of nationally representative adults residing in the UK. Our study included a relatively equal proportion of women and men from socio-economically diverse backgrounds. Because we did not rely on the clinical diagnosis to identify people who were at an increased risk for ADHD, but instead used a continuous measure of polygenetic liability to ADHD, our results are unlikely to be affected by sex biases in the referral practices, delaying diagnosing females with ADHD, which consequently may be translated into research [42]. Although previous research alluded that ADHD affects life expectancy differently in men and women [2], the present study has addressed the gap in the current knowledge of sex differences in ADHD-related research, which have been largely neglected [43–45].

Even though no participants reported a diagnosis of ADHD, the condition is known to be difficult to diagnose. Given there is evidence that around 4% of adults have ADHD [46], the absence of ADHD cases in ELSA may imply underdiagnosis. The survival bias effect could have attenuated association of a polygenic predisposition to ADHD and all-cause mortality. Specifically, a higher proportion of people with a high ADHD manifestation could have experienced mortality and hence were under-represented in the ELSA cohort. This in turn may have led to an underestimation of the associations between PGSs and all-cause mortality. Even though PGSs can be seen as unconfounded proxies for the life-time predisposition to ADHD, a gene-environmental correlation may still be present, which in turn may influence the mortality risk in the general population. The low generalisability of genetic studies across populations is noteworthy [21]. This is because the construction of PGSs is mainly dependent on the availability of the summary statistics from GWASs, which are currently predominately based on European participants [21]. Similarly, because PGSs are built on GWAS, they may be restricted by the same limiting factors that are inherent to GWASs, such as being unable to capture rare variants, poorly tagged or multiple independent variants, gene-by-gene interactions and gene-environment correlation [47]. To minimise chances of collider bias affecting our findings [48], all covariates that were included in the models were set at birth; on the other hand, however, we did not adjust the confounding effect of others factors, such as smoking and educational attainment on the mortality risk. Nonetheless, potentially mediating effects of these factors on the nexus of ADHD and mortality could be assessed in future studies. Finally, the reported associations may be influenced by the other correlated traits, which may be independently associated with all-cause mortality.

## Conclusion

Polygenic predisposition to ADHD confers increased risk for all-cause mortality in the general population of adults aged 50 years old and onwards. Our results further suggest that to fully capture the genetic risk of ADHD, it is imperative to incorporate genetic information contained in traits with an overlapping molecular basis.

## Supplementary Information

**Additional file 1: Figure S1.** Depicts distribution of 10 principal components once 65 individuals with ancestral admixture were removed from the sample. **Figure S2.** Depicts every step of quality control and assurance that was undertaken in preparation of the genetic data for the analyses in the ELLSA study. **Table S1.** Comparisons between the ELSA participants who were included in the analyses and those who were excluded. **Table S2.** The rate of mortality at each year of follow-up. **Table S3.** An overview of the summary of full QC procedure employed in the ELSA study and how many variants and/or participants were lost at each step. **Table S4.** Cox regression analyses highlighting associations between PGS-ADHD_*P*T=1_ and risk for all-cause mortality during a follow-up period. **Table S5.** Results of a global test for violation of proportional hazards assumption for the fully adjusted Cox models.

## Data Availability

The English Longitudinal Study of Ageing (ELSA) was developed by a team of researchers based at University College London, the Institute for Fiscal Studies and the National Centre for Social Research. The datasets generated and/or analysed during the current study are available in UK Data Services and can be accessed at: https://discover.ukdataservice.ac.uk. No administrative permissions were required to access these data.

## Abbreviations

1000GP: 1000 Genomes Project
ADHD: Attention deficit hyperactivity disorder
CI: Confidence interval
ELSA: English Longitudinal Study of Ageing
GWAS: Genome-wide association study
HR: Hazard ratio
LD: Linkage disequilibrium
MAF: Minor allele frequency
PC: Principal component
PGS: Polygenic score
pT: *P*-value threshold
SNP: Single nucleotide polymorphism
MTAG: Multi-Trait Analysis of GWAS
