# Supplementary material for: High polygenic predisposition for ADHD and a greater risk of all-cause mortality: a large population-based longitudinal study
Source: BMC Med. 2022 Feb 23;20:62. doi: 10.1186/s12916-022-02279-3 (PMC8864906; doi:10.1186/s12916-022-02279-3)
Supplement: Supplementary file 1 — Additional file 1: Figure S1. Depicts distribution of 10 principal components once 65 individuals with ancestral admixture were removed from the sample. Figure S2. Depicts every step of quality control and assurance that was undertaken in preparation of the genetic data for the analyses in the ELLSA study. Table S1. Comparisons between the ELSA participants who were included in the analyses and those who were excluded. Table S2. The rate of mortality at each year of follow-up. Table S3. An overview of the summary of full QC procedure employed in the ELSA study and how many variants and/or participants were lost at each step. Table S4. Cox regression analyses highlighting associations between PGS-ADHDPT=1 and risk for all-cause mortality during a follow-up period. Table S5. Results of a global test for violation of proportional hazards assumption for the fully adjusted Cox models. [file 12916_2022_2279_MOESM1_ESM.docx]

**Figure S1.** Depicts distribution of 10 principal components once 65 individuals with ancestral admixture were removed from the sample.


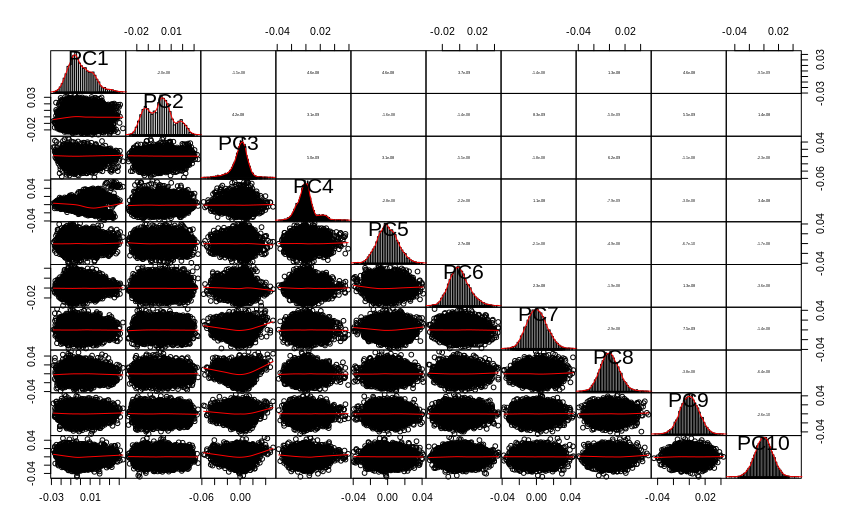


**Figure S2.** Depicts every step of quality control and assurance that was undertaken in preparation of the genetic data for the analyses in the ELLSA study.

**Table S1.**  Comparisons between the ELSA participants who were included in the analyses and those who were excluded

|  |  |  | **Excluded (n=4955)** | **Included (n=7133)** |  |
| --- | --- | --- | --- | --- | --- |
|  |  |  | Mean (SD) / n(%) | Mean (SD) / n(%) | Statistics |
| *Socio-demographic characteristics* | |  |  |  |  |
|  | Age (years) |  | 66.4 (10.3) | 64.7 (9.6) | *t*=8.8, df=11986, *p*<0.001 |
|  | Gender (male) |  | 2147 (44.5) | 3294 (46.2) | x^2^=3.4, df=1, *p*=0.065 |
|  | Education (years) |  | 13.2 (3.7) | 13.7 (3.8) | *t*=-7.1, df=10941, *p*<0.001 |
|  | Married |  | 1759 (36.4) | 2227 (31.2) | x^2^=35.0, df=1, *p*<0.001 |
| Accumulated wealth | |  |  |  | x^2^=88.2, df=2, *p*<0.001 |
|  | High |  | 1338 (29.2) | 2453 (35.3) |  |
|  | Intermediate |  | 1484 (32.4) | 2382 (34.3) |  |
|  | Low |  | 1762 (38.4) | 2109 (30.4) |  |
|  |  |  |  |  |  |
| *Comorbid health issues* | |  |  |  |  |
|  | Limiting health conditions |  | 1902 (39.7) | 2313 (32.4) | x^2^=65.8, df=1, *p*<0.001 |
|  | Depression (score ≥4) |  | 563 (18.8) | 800 (13.8) | x^2^=38.3, df=1, *p*<0.001 |
|  |  |  |  |  |  |
| *Behavioural outcomes* | |  |  |  |  |
|  | Currently a smoker |  | 800 (16.6) | 1047 (14.7) | x^2^=8.0, df=1, *p*=0.005 |

**Table S2.** The rate of mortality at each year of follow-up

| **Year of participation in the study** | **Mortality** | |
| --- | --- | --- |
|  | **n (%)** | **Age, Median (IQR)** |
| Year 1 | 69 (1.0) | 74.0 (16.0) |
| Year 2 | 91 (1.3) | 77.0 (10.5) |
| Year 3 | 93 (1.3) | 77.0 (15.0) |
| Year 4 | 121 (1.7) | 75.0 (13.0) |
| Year 5 | 107 (1.5) | 75.0 (16.0) |
| Year 6 | 145 (2.0) | 76.0 (12.0) |
| Year 7 | 159 (2.2) | 76.0 (15.0) |
| Year 8 | 152 (2.1) | 73.5 (12.0) |
| Year 9 | 157 (2.2) | 75.0 (11.0) |
| Year 10 | 141 (2.0) | 72.0 (13.0) |
| Year 11 | 168 (2.4) | 72.0 (12.3) |
| Year 12 | 142 (2.0) | 73.5 (12.0) |
| Year 13 | 169 (2.4) | 71.0 (12.0) |
| Year 14 | 64 (0.9) | 73.0 (11.5) |

IQR – interquartile range (0.75-quantile minus 0.25-quantile)

**Table S3.** An overview of the summary of full QC procedure employed in the ELSA study and how many variants and/or participants were lost at each step

| Quality Control steps in ELSA | | | |
| --- | --- | --- | --- |
| *Lost due to SNP-based QC* | | *n* | % |
|  | Missing SNPs (0.02) | 41614 | 1.87 |
|  | Autosomal SNPs | 48578 | 2.18 |
|  | MAF 0.01 | 759972 | 34.07 |
|  | Update rsids | 2284 | 0.10 |
|  | HWE (0.0001) | 6079 | 0.27 |
|  |  |  |  |
|  | *Total removed* | *858527* | *38.49* |
|  | *Total remaining* | *1372240* | *61.51* |
|  |  |  |  |
| *Lost due to Individual-based QC* | |  |  |
|  | Missingness (0.02) | 39 | 0.53 |
|  | Heterogeneity | 76 | 1.03 |
|  | Sex discordance | 5 | 0.07 |
|  | Ancestry outliers | 64 | 0.86 |
|  | Relatedness/Duplicates | 5 | 0.07 |
|  | Unique IDs are not present | 41 | 0.50 |
|  |  |  |  |
|  | *Total removed* | *229* | *3.09* |
|  | *Total remaining* | *7183* | *96.91* |

HWE, Hardy-Weinberg equilibrium; MAF, minor allele frequency; SNP, single nucleotide polymorphisms

**Table S4**. **Cox regression analyses highlighting associations between PGS-ADHD*_P_*_T=1_ and risk for all-cause mortality during a follow-up period**

| **Polygenic scores for ADHD** | **The whole sample** | | | | **Men** | | | | **Women** | | | |
| --- | --- | --- | --- | --- | --- | --- | --- | --- | --- | --- | --- | --- |
|  | **HR** | **95% CI** | ***p*** | **R^2^** | **HR** | **95% CI** | ***p*** | **R^2^** | **HR** | **95% CI** | ***p*** | **R^2^** |
| *Participants all ages* |  |  |  |  |  |  |  |  |  |  |  |  |
| PGS-ADHD*_P_*_T=1_ | 1.05 | 1.00-1.10 | 0.047 | 0.00% | 1.07 | 1.00-1.14 | 0.047 | 0.09% | 1.03 | 0.96-1.10 | 0.416 | 0.01% |
| PGS-ADHD _PT=0.3_ | 0.96 | 0.76-1.22 | 0.759 | 0.00% | 1.06 | 0.99-1.13 | 0.073 | 0.07% | 1.03 | 0.96-1.10 | 0.366 | 0.02% |
| PGS-ADHD _PT=0.1_ | 0.95 | 0.85-1.06 | 0.347 | 0.01% | 1.05 | 0.98-1.12 | 0.152 | 0.05% | 1.02 | 0.95-1.09 | 0.612 | 0.01% |
| PGS-ADHD _PT=0.05_ | 0.95 | 0.87-1.03 | 0.231 | 0.02% | 1.03 | 0.96-1.10 | 0.385 | 0.02% | 1.02 | 0.95-1.09 | 0.661 | 0.00% |
| PGS-ADHD _PT=0.01_ | 0.92 | 0.87-0.98 | 0.010 | 0.07% | 0.97 | 0.91-1.04 | 0.384 | 0.02% | 0.99 | 0.93-1.06 | 0.805 | 0.00% |
| PGS-ADHD _PT=0.001_ | 0.94 | 0.89-0.99 | 0.013 | 0.07% | 0.95 | 0.89-1.01 | 0.088 | 0.07% | 0.99 | 0.92-1.06 | 0.699 | 0.00% |
|  |  |  |  |  |  |  |  |  |  |  |  |  |
| *Participants aged 50-75y at* |  |  |  |  |  |  |  |  |  |  |  |  |
| PGS-ADHD*_P_*_T=1_ | 1.08 | 1.02-1.15 | 0.012 | 0.00% | 1.11 | 1.02-1.21 | 0.014 | 0.19% | 1.05 | 0.95-1.15 | 0.357 | 0.02% |
| PGS-ADHD _PT=0.3_ | 0.91 | 0.67-1.26 | 0.582 | 0.01% | 1.10 | 1.01-1.20 | 0.030 | 0.15% | 1.05 | 0.96-1.16 | 0.310 | 0.03% |
| PGS-ADHD _PT=0.1_ | 0.94 | 0.81-1.08 | 0.371 | 0.01% | 1.08 | 1.00-1.18 | 0.061 | 0.11% | 1.03 | 0.94-1.13 | 0.516 | 0.01% |
| PGS-ADHD _PT=0.05_ | 0.93 | 0.83-1.03 | 0.173 | 0.03% | 1.06 | 0.98-1.16 | 0.167 | 0.06% | 1.02 | 0.93-1.12 | 0.681 | 0.01% |
| PGS-ADHD _PT=0.01_ | 0.88 | 0.82-0.96 | 0.003 | 0.13% | 0.97 | 0.89-1.06 | 0.474 | 0.02% | 0.99 | 0.90-1.09 | 0.790 | 0.00% |
| PGS-ADHD _PT=0.001_ | 0.91 | 0.85-0.97 | 0.005 | 0.12% | 0.94 | 0.86-1.02 | 0.130 | 0.07% | 0.97 | 0.88-1.06 | 0.485 | 0.01% |

ADHD, Attention Deficit Hyperactivity Disorder; HR, hazard ratio; CI, confidence intervals

Model was adjusted for baseline age, gender, interaction between age, gender and first 4 PCs to adjust for genetic ancestry; to capture non-linear effects of aging, we included age^2^ and age^3^ as covariates.

**Table S5**. Results of a global test for violation of proportional hazards assumption for the fully adjusted Cox models.

|  | **The whole sample** | **Separate for each gender** | |
| --- | --- | --- | --- |
|  |  | **Men** | **Women** |
| Model | *p*=0.352^1^ | *p*=0.764^2^ | *p*=0.851^2^ |

*P*-values above 0.05 show that proportional hazards assumption cannot be ruled out

^1^model passed the global test for proportional hazards assumption, however, by factor analysis revealed that the gender factor violated the test. Examination of the Schoenfield residuals showed that impact of gender was decreasing with time which indicated that a separate analysis for both genders could deliver better fitted models. ^2^Other factors did not violate PH assumptions and global test has higher p-values for separate models for each gender.
